# Rethinking the classification of acute status epilepticus: structural brain vs. systemic etiologies

**DOI:** 10.3389/fneur.2026.1763224

**Published:** 2026-01-20

**Authors:** Sophie Xhepa, Paola Gullino, Pia De Stefano

**Affiliations:** 1 Neurology Unit, Department of Clinical Neurosciences, University Hospital of Geneva, Geneva, Switzerland; 2 Neuro-Intensive Care Unit, Department of Intensive Care, University Hospital of Geneva, Geneva, Switzerland; 3 Neurology Unit, University Hospital of Siena, Siena, Italy; 4 Department of Clinical Neuroscience, University of Geneva, Geneva, Switzerland

**Keywords:** acute status epilepticus, classification, epileptogenesis, etiology, metabolic disturbances, pathophysiology, structural brain injury, systemic causes

## Abstract

Status epilepticus (SE) is a life-threatening neurological emergency whose modern conceptualization employs a multidimensional framework encompassing semiology, etiology, electroencephalographic features, and age. Among these, the etiological dimension is central, as it captures the underlying pathophysiology and critically informs both acute management and long-term prognosis. Current classifications define acute SE by its temporal proximity to a central nervous system insult, yet this approach groups heterogeneous mechanisms under a single category. Acute SE arising from structural brain injury involves downstream receptor and ionic dysfunction driven by inflammation, neuronal loss, glial activation, and network remodeling. In contrast, systemic etiologies, such as electrolyte disturbances, toxic exposures, or withdrawal states, primarily reflect transient disruptions in excitatoryinhibitory balance or ionic homeostasis. These mechanistic distinctions translate into meaningful clinical differences: structural causes are associated with higher mortality, greater seizure recurrence, and an increased risk of subsequent epilepsy. A more refined etiological classification distinguishing structural from systemic causes could better capture variations in pathophysiology, treatment response, and long-term outcomes. Such a framework may also guide decisions regarding the duration and intensity of antiseizure medication (ASM) therapy, a domain in which evidence remains limited. Whether early and sustained ASM treatment after SE due to structural brain injury can modify epileptogenesis is unknown, as no randomized controlled trials have directly addressed this question. Clarifying etiological subgroups may therefore facilitate targeted clinical and translational studies, improve prognostication, and ultimately support the development of interventions to prevent the transition from acute SE to chronic epilepsy.

## Introduction: the acute status epilepticus etiology

Status epilepticus (SE) is a common medical emergency associated with significant morbidity and mortality (1), a heterogeneous condition resulting from failed seizure terminating mechanisms or from pathological processes that sustain seizure activity beyond a critical threshold (t1). Ongoing seizure activity can progress to a second stage (t2), when long-term consequences arise (2).

Over the past decades, the conceptualization and definition of SE have evolved substantially (3–6) culminating in the 2015 ILAE framework (2), which proposes a classification based on four axes: semiology, etiology, electroencephalographic correlates, and age. Among these, the etiological axis is particularly relevant as it reflects pathophysiological mechanisms and directly informs both acute management and long-term prognosis. Based on the etiological axis, SE may be categorized as acute, remote, progressive, associated with a defined electroclinical syndrome, or of unknown origin.

Acute etiology is generally the category that receives the most attention and discussion, as it is the most common in the hospital setting, especially in the Intensive Care Unit (7).

A clear timeline is not defined in the ILAE classification of acute SE; however, acute SE represents the most severe manifestation of acute symptomatic seizures (8). These seizures occur in close temporal association with an acute insult to the central nervous system, arising from systemic disturbances (metabolic, toxic, or withdrawal related) or from cerebral structural disorders (traumatic, vascular, or infectious) (8). The accepted temporal window varies by etiology: up to 24 h for systemic causes and typically 7 days for traumatic or vascular structural insults. Nevertheless, recent risk-stratification studies in the context of stroke (9) indicate that in a substantial proportion of cases the long-term risk of unprovoked seizures exceeds the 60% threshold used for the operational diagnosis of epilepsy, thereby challenging a strictly temporal classification.

This distinction is further blurred in infectious and inflammatory conditions, in which seizures are considered acute symptomatic as long as active infection/inflammation persists. In autoimmune encephalitis, the ILAE has proposed response to immunotherapy as a conceptual criterion to distinguish acute symptomatic seizures from epilepsy (10). However, this framework remains challenging to apply in clinical practice, since long-term epilepsy risk appears to be largely influenced by the underlying pathophysiology—particularly the nature of the immune response (antibodies against extracellular versus intracellular antigens) and the extent of immune-mediated structural brain injury—rather than by temporal criteria alone, and by the lack of both validated therapies and reliable biomarkers to accurately define the resolution of active inflammation (11).

The 2015 ILAE Task Force on Classification of Status Epilepticus does not further subdivide acute causes into subcategories; the goal is to provide a practical framework for diagnosis and management, in those etiologies that share temporal proximity and potential reversibility, as their identification is critical for guiding acute treatment and prognosis (2).

However, acute SE does not represent a homogeneous entity: therapeutic strategies differ depending on whether the precipitating factor can be rapidly reversed (e.g., systemic insults), or whether it reflects an acute structural brain lesion, potentially permanent.

In recent years, a new classification system for acute SE has been proposed (12) organizing acute SE etiologies into four distinct subgroups:

1. Acute triggers in individuals with pre-existing epilepsy, such as withdrawal or low levels of antiseizure medications (ASMs), febrile illness, or sleep deprivation.
2. Acute primary CNS (central nervous system) pathology, defined as new brain injuries or diseases directly affecting the CNS.
3. Acute secondary CNS pathology, involving non-CNS conditions that secondarily affect the brain, such as systemic or metabolic disturbances.
4. Acute toxic causes, referring to external substances or drugs, such as alcohol withdrawal or exposure to toxins/drugs, triggering SE.

The authors showed that these four etiological groups were associated with distinct clinical outcomes and seizure recurrence risks both higher for the acute-primary CNS etiologies and lower for the acute triggering factors highlighting their clinical and prognostic value (12, 13).

The classification framework proposed by Lattanzi et al. was developed excluding post-anoxic SE. This reflects the recognition that post-anoxic SE represents a clinically and prognostically distinct entity with markedly poorer outcomes and different pathophysiological mechanisms. Accordingly, prognostic models and classification schemes derived from non-hypoxic–ischemic etiologies should be interpreted with caution when applied to the post-cardiac arrest patients subgroup (13).

## Epidemiology of acute status epilepticus

Epidemiological data on acute SE remain limited. Before the 2015 ILAE classification, most studies did not distinguish between acute and non-acute etiologies, making incidence and outcome data unreliable. With the updated criteria, recent studies report more accurate measures of incidence, distribution of etiological subtypes, semiology and outcome, clarifying the distinct features and burden of acute SE within the SE spectrum.

The incidence of SE varies among studies, ranging from 15.6 to 36.1/100,000 (14, 15) with a significant proportion of patients, 24.8–68.7% (15), reporting acute etiologies. Among those, acute cerebrovascular disease, brain tumors, infectious, metabolic, alcohol-related mechanisms, and toxic disorders are the leading causes (14). An interesting meta-analysis of 43 studies found that acute symptomatic etiology accounted for the largest attributable fraction of SE cases (16).

In terms of SE semiology, if nonconvulsive SE in coma is more frequent in the acuteprimary CNS subcategory (12), convulsive SE is more frequent in the withdrawal/low ASM and acute-toxic subcategories (17). As expected, mortality risk is higher for nonconvulsive SE than convulsive SE (18).

Overall, in-hospital mortality in SE shows wide variability, with reported rates between 4.6 and 39% (19, 20), while acute symptomatic causes exhibit particularly high mortality rates, ranging from 53.1 to 73.7% (12, 14).

When examined by acute etiologic subcategory, mortality of acute SE varies substantially. One study reported rates of 30.6% for primary CNS causes, 32% for secondary causes such as metabolic disturbances, and 7.1% for acute toxic etiologies or acute precipitants in patients with preexisting epilepsy (12). Another study (21) found mortality rates of 10% for acute cerebrovascular etiologies, 6% for traumatic brain injury, and 13% for metabolic derangements. In both analyses, metabolic abnormalities were grouped within broad metabolic categories, including electrolyte disturbances associated with systemic diseases such as cirrhosis, limiting the ability to assess their specific contribution to SE-related mortality.

Although multiple etiologic factors frequently coexist in acute SE, episodes are commonly attributed to the factor considered the predominant driver of the acute event. This pragmatic approach simplifies classification but may incompletely capture clinical reality, in which acute structural brain injuries often coexist with systemic precipitants and with remote or progressive conditions, thereby complicating etiologic attribution and prognostic stratification in routine practice (7, 12).

## Pathophysiology and epileptogenesis: structural brain versus systemic etiologies

Acute SE is driven by immediate and potentially reversible pathophysiological mechanisms, whereas remote symptomatic/progressive SE arises from preexisting and irreversible alterations of the cerebral network.

**Figure 1:**
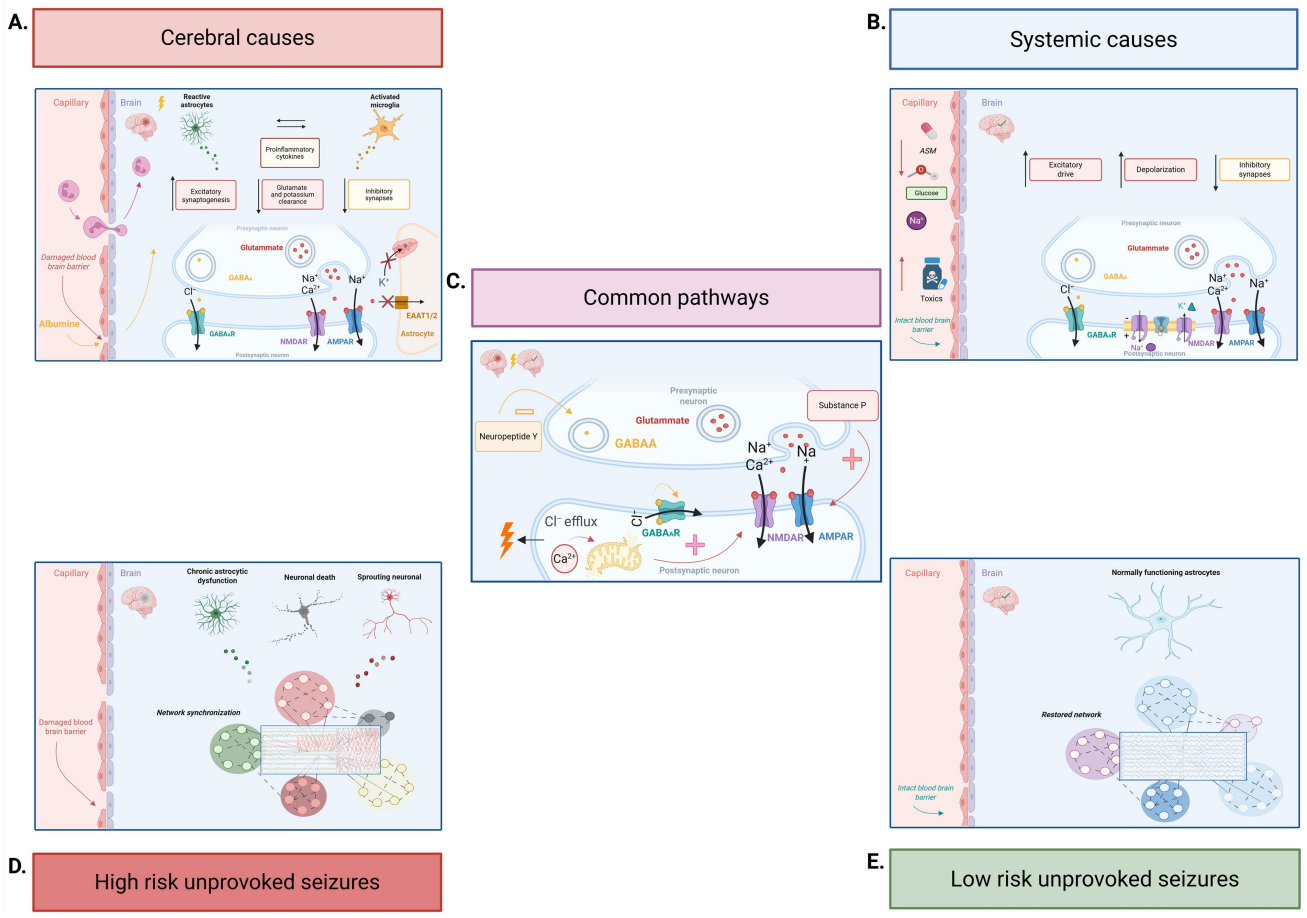
Pathophysiological pathways of acute status epilepticus. Created with BioRender.com. **(A)** Pathways associated with structural brain etiologies. **(B)** Pathways associated with systemic etiologies. **(C)** Shared pathways common to both etiological categories. **(D)** Consequences of acute status epilepticus due to structural brain causes. **(E)** Consequences of acute status epilepticus due to systemic causes.

As illustrated in Figure 1 acute SE stems from heterogeneous intracranial (Figure 1A) and extracranial causes (Figure 1B) that initiate and shape the early phase through distinct mechanisms, they use a shared ionic, molecular, and network pathways that enable the transition to a self-sustaining state (22, 23). This unified pathophysiological framework (Figure 1C) reflects a progressive disruption of inhibitory-excitatory balance across distinct physiopathological layers.

Acute SE involves rapid internalization of GABA receptors and synaptic recruitment of NMDA and AMPA receptors, shifting synaptic transmission toward hyperexcitation (24–26).

Ion homeostasis deteriorates in parallel: downregulation of KCC2, leads to chloride accumulation, while NMDA-mediated calcium influx further destabilizes membrane potentials. As GABAergic inhibition depends on chloride gradients, this collapse renders GABA-receptor activation increasingly depolarizing, reinforcing seizure selfsustainment (26).

These ionic disturbances place a high metabolic demand on inhibitory interneurons and disrupt neuropeptidergic signaling; substance P, upregulated by sustained neuronal activity, enhances glutamate release and strengthens excitatory drive, whereas neuropeptide Y, normally released by inhibitory interneurons to restrain glutamate release, declines as these interneurons become metabolically compromised. The imbalance between excitatory and inhibitory neuropeptides removes a key modulatory brake and further stabilizes the ictal state.

Converging receptor and ionic abnormalities lead to mitochondrial calcium overload, impaired buffering, oxidative stress, and reactive oxygen species production, promoting neuronal injury and transcriptional changes that maintain network hyperexcitability.

With increasing duration, acute SE can induce blood–brain barrier (BBB) dysfunction and glial activation; however, the magnitude of this effect is largely determined by the presence and extent of preexisting neuronal injury. The barrier disruption amplifies neuroinflammation, impairs glutamate and potassium clearance, and facilitates the persistence of ictal activity (25–28).

In acute SE, network reverberation emerges dynamically rather than through preexisting epileptogenic pathways, as is typical in remote forms (27). It is characterized by a transition from self-limited ictal events to widespread, persistent, and highly synchronized neuronal firing. The hippocampus, among the most seizure-prone structures, is generally recruited early and frequently acts as a central hub for propagation, with subsequent involvement of parahippocampal regions, neocortex, thalamus, and additional subcortical areas. As the episode progresses, large-scale cortico-cortical and cortico-subcortical re-entrant loops become engaged, promoting bilateral synchronization (22, 29).

Acute SE due to a *structural brain insult* (Figure 1A) (such as stroke, trauma, or CNS infection) is driven by BBB dysfunction that triggers a robust inflammatory response promoting maladaptive receptor trafficking, ionic shifts, and ultimately mitochondrial dysfunction and neuronal death (23, 30). Loss of barrier integrity permits albumin extravasation, which rapidly activates astrocytes and microglia through neuroinflammatory pathways—notably TGF-*β* signaling—thereby lowering seizure threshold, enhancing excitatory synaptogenesis, and facilitating network synchronization and positive feedback loops that perpetuate excitotoxicity and neuronal injury.

Astrocyte activation is sustained by underlying structural damage, inducing persistent gliosis, impaired glutamate and potassium clearance, and the release of cytokines and chemokines that directly modulate neuronal excitability. This process increases overall seizure burden and fuels the underlying vicious cycle (23, 31, 32).

SE arising from *systemic insult* (Figure 1B) is driven by non-primary cerebral disturbances that globally alter neuronal excitability. These perturbations act either by directly changing neuronal membrane potential or by shifting the balance of synaptic transmission, reducing inhibitory GABAergic drive, enhancing excitatory glutamatergic tone, or both.

One example is the electrolyte disturbances that precipitate SE when ionic imbalance, most commonly involving sodium, potassium, calcium, or chloride, becomes sufficiently severe to disrupt membrane potentials and impair synaptic signaling. For example, hyponatremia lowers the threshold for neuronal depolarization, whereas hyperkalemia and hypocalcemia destabilize membrane potentials and facilitate sustained firing; the resulting instability promotes persistent, hypersynchronous excitatory activity that overwhelms endogenous inhibitory mechanisms (28, 33, 34).

On the other hand, intoxication with proconvulsant agents, including cocaine and amphetamines, can directly enhance excitatory transmission through NMDA and AMPA receptor activation. Conversely, substances such as penicillin and related βlactams exert a proconvulsant effect by antagonizing GABAergic inhibition, lowering seizure threshold and favoring persistence of ictal activity (17).

Withdrawal syndromes represent another major systemic trigger. Abrupt cessation of alcohol or benzodiazepines removes chronic GABAergic potentiation, unmasking a compensatory upregulation of excitatory glutamatergic receptors and resulting in a net increase in excitatory drive. Similarly, sudden discontinuation of antiseizure medications can precipitate SE through acute loss of pharmacological control over neuronal excitability, with reduced GABAergic inhibition and/or enhanced glutamatergic transmission (22, 30, 35, 36).

Epileptogenesis after acute SE depends on whether the initiating event produces enduring structural alterations in neuronal and glial networks. When it occurs in the setting of a structural brain injury, the combination of neuronal loss, astrocytic and microglial activation, and persistent BBB dysfunction initiates a cascade of maladaptive remodeling. Focal neuronal death disrupts inhibitory microcircuits, creating gaps that are filled by aberrant axonal sprouting and rewiring of excitatory pathways. Chronic astrocytic dysfunction, marked by impaired glutamate and potassium buffering and continued release of proinflammatory mediators, sustains a hyperexcitable extracellular milieu. These processes jointly alter synaptic connectivity, shift network dynamics toward synchronization, and establish circuits capable of generating recurrent spontaneous seizures (Figure 1D) (23, 30, 37).

In contrast, systemic causes of SE rarely produce the structural or glial alterations needed for lasting epileptogenesis; synaptic and network function return to baseline without the persistent astrocytic activation, BBB disruption, or microcircuit remodeling that follow structural injury (Figure 1E). Even when prolonged seizures from systemic causes trigger brief inflammatory responses, these are generally insufficient to induce the enduring synaptic and network changes required for chronic epilepsy (13, 17).

## Role of treatment and risk of epilepsy

International guidelines recommend a uniform pharmacologic approach to the treatment of acute SE, irrespective of the underlying cause, reflecting the priority of rapid seizure termination (24). Identification and management of the precipitating etiology should occur in parallel with ASM administration to optimize clinical outcomes. To date, no evidence indicates that the choice, sequence, or dosing of ASMs should be modified solely on the basis of etiology. The rationale for an uniform pharmacologic approach is that available treatments act on the common pathways that allow SE to become self-sustaining, rather than on the acute underlying process that triggers it. There is no evidence from clinical studies comparing the efficacy of.

ASMs—of first, second or third classes—according to different acute etiologies of SE. The selection of a specific agent is therefore guided primarily by the timing of treatment initiation and by the patient’s response to preceding therapies, rather than by the underlying cause (24).

The decision to continue long-term antiseizure therapy after acute SE should be individualized on the basis of the underlying etiology and the estimated risk of seizure recurrence (38). While the risk of relapse is high in SE associated with a structural brain insult, it is negligible when SE arises from a systemic disturbance (39). This distinction is expected: in SE due to a systemic acute etiology, the provoking factor is typically reversible and can be rapidly corrected, removing the need for long-term antiseizure therapy. Conversely, in structural brain etiologies, the underlying lesion is often not reversible in the acute phase, except in select cases such as subdural hematoma or brain abscess, and may progress to a permanent cortical abnormality with epileptogenic potential. As noted above, these two conditions also differ fundamentally in their pathophysiology: systemic etiologies primarily involve a transient imbalance in excitatory-inhibitory neurotransmission or ionic homeostasis, whereas acute intracerebral causes reflect receptor or ionic dysfunction secondary to inflammation, neuronal loss, glial activation, and network reorganization.

Typically, long-term ASM is initiated after a first unprovoked seizure when the estimated risk of relapse is ≥60%, in line with the diagnostic threshold for epilepsy (40). In acute SE, however, this criterion is not met, as the event does not constitute an unprovoked seizure, even in the presence of a brain “epileptogenic” lesion. But, at the same time, the risk of developing epilepsy after an acute SE may reach, in specific etiologies and with associated factors (41–43) 81–88%, far exceeding the 60% threshold used in the International League Against Epilepsy. A correlation between the duration of the SE and the subsequent development of epilepsy has also been observed (43).

These clinical scenarios, therefore, challenge the current definition of epilepsy.

This important evidence on risk prediction and understanding of long-term epilepsy has emerged in the last years from the stroke and traumatic brain injury populations.

### Acute SE in the stroke population

The presence of electrographic SE after stroke is a strong predictor of a markedly increased long-term risk of developing epilepsy. The 10-year risk of post-stroke epilepsy in patients with acute SE reaches 81–88% and is much higher than the risk after short acute symptomatic seizures (40%) or no acute symptomatic seizures (13%) (44). This elevated risk is independent of other clinical factors such as age, stroke severity, location, and etiology, and is robust across large multicenter cohorts and replication studies. The SeLECT 2.0 prognostic model incorporates the type of acute symptomatic seizure for risk stratification, assigning additional weight to acute SE and thereby identifying patients at very high risk of post-stroke epilepsy (44). A subsequent refinement further improved prognostic accuracy by replacing baseline stroke severity with neurological severity assessed at 72 h after stroke onset, underscoring the relevance of residual post-treatment injury for epileptogenesis (9).

Similarly, in hemorrhagic stroke, acute SE occurring within the first week, rather than short seizures, has been shown to independently predict all-cause mortality in addition to the development of post-stroke epilepsy, highlighting the distinct prognostic implications of seizure subtype across stroke etiologies (45).

The literature also notes that SE after stroke is associated with high mortality, and most patients with this presentation receive ASMs, though the optimal duration of therapy remains uncertain. Continuous EEG monitoring in the acute phase can further refine risk stratification, but the presence of SE itself is the most potent clinical predictor of epileptogenesis in this context (46). Systematic reviews and metaanalyses indicate that, despite ASM use, seizure recurrence rates remain high in the post-stroke population, with recurrence rates around 25% and substantial heterogeneity in outcomes (47–49). There is a lack of high-quality randomized controlled trials specifically addressing the impact of long-term ASM therapy after SE on the development of epilepsy (47, 49, 50). Routine primary prophylaxis with ASMs is not recommended except in select high-risk cases, such as severe cortical involvement or hemorrhagic stroke (50). Secondary prophylaxis, initiating ASMs after an unprovoked seizure, remains standard, but the optimal duration of therapy after status epilepticus is not established (49).

### Acute SE in the traumatic brain injury population

Similarly to post-stroke epilepsy, the risk of posttraumatic epilepsy (PTE) is high in patients with severe TBI and those who experience early seizures, particularly SE, with cumulative incidence rates of PTE reaching up to 32% at 15 years in severe TBI cohorts (51, 52). Continuous EEG monitoring has revealed that nonconvulsive SE is underdiagnosed and may contribute to secondary injury and hippocampal atrophy, further increasing the risk of chronic epilepsy (53). The American College of Surgeons, in its guideline, identifies early seizures, especially those with delayed onset and SE, as major risk factors for PTE and recommends close monitoring in these patients (54). The development of PTE is multifactorial, but SE is a clear marker of high risk and poor prognosis for long-term neurological outcomes (51). Multiple high-quality systematic reviews and network meta-analyses confirm that while phenytoin, levetiracetam, and carbamazepine are effective in reducing early (within 7 days) posttraumatic seizures, no antiseizure medication regimen, regardless of timing or duration, has been shown to reduce the incidence of late posttraumatic epilepsy (51, 55, 56). Prolonged or unnecessary use of antiseizure medications beyond 7 days is not recommended and may be associated with adverse effects and impaired recovery (51).

### Emerging biomarkers of epileptogenesis

Given the difficulty in clinically defining and measuring epileptogenesis, increasing attention has been directed toward emerging biomarkers that may support risk stratification and disease monitoring. A structured roadmap for biomarker discovery and validation has been proposed to harmonize methodologies and facilitate translation into clinical research (57). Within this framework, post-SE represents a relevant epileptogenic model in which candidate biomarkers, particularly those reflecting neuroinflammatory and glial responses, may help stratify long-term epilepsy risk across different acute cerebral and systemic etiologies, while also providing pathophysiological insight into injury-related network remodeling (58). If validated, such biomarkers could also inform the rational selection of patients for targeted preventive or disease-modifying interventions, including primary prevention strategies for epilepsy.

However, available data are still evolving, and the role of these approaches in individual-level prediction requires further validation.

## Prevention treatment

It remains unclear whether early and prolonged ASM therapy after SE following acute structural brain insult can modify the long-term risk of developing epilepsy. No randomized controlled trials have directly addressed this question, and the current consensus is that ASMs are indicated for acute management and, in selected cases, for secondary prevention, but their role in preventing epileptogenesis is unproven. No therapeutic agent has yet shown proven efficacy in preventing epileptogenesis after acute SE in patients without a prior history of epilepsy (38, 59).

Experimental studies have suggested potential antiepileptogenic effects for agents such as levetiracetam, losartan, and anti-inflammatory drugs, but these findings have not translated into clinical benefit, and no agent is currently recommended (60–62).

Eslicarbazepine, perampanel, low-dose levetiracetam, and biperiden have been proposed as potential antiepileptogenic agents because they modulate key pathways involved in epileptogenesis, including neuronal hyperexcitability, maladaptive synaptic plasticity, excitatory neurotransmission, and neuroinflammation (61, 63–67).

Several pharmacologic trials are currently investigating whether early intervention with these or other compounds can reduce the risk of epilepsy after acute brain injury, particularly in post-stroke and traumatic brain injury populations. These studies remain ongoing, and no conclusive evidence yet supports the routine use of any agent for the prevention of epileptogenesis in clinical practice (54, 68–70).

## Conclusion

The classification of SE relies on a multidimensional framework that integrates etiology, electroencephalographic features, and age. Among these axes, etiology is particularly relevant because it reflects the underlying pathophysiology. At present, however, the heterogeneous causes of acute SE are grouped primarily by their temporal relationship to SE onset rather than by their mechanistic differences. In acute intracranial causes involve receptor or ionic dysfunction as a downstream effect of inflammation, neuronal loss, glial activation, and network remodeling. On the contrary, in systemic etiologies, such as electrolyte disturbances, toxic exposures, or withdrawal, the initiating factor is an imbalance in excitatory–inhibitory neurotransmission or ionic homeostasis. These distinctions are mirrored in epidemiologic data, with structural causes associated with higher mortality and an increased risk of subsequent epilepsy. These observations suggest that the classification of acute SE may benefit from a clearer distinction between structural brain etiologies and systemic causes. A framework that accounts for these differences would more accurately reflect variations in pathophysiology, treatment response, and long-term outcomes, and could help inform decisions regarding the duration and intensity of antiseizure therapy. This is of utmost importance, as it remains unclear whether early and prolonged ASM therapy after SE following brain insult can modify the long-term risk of developing epilepsy. No randomized controlled trials have directly addressed this question, and the current consensus is that ASMs are indicated for acute management and secondary prevention, but their role in preventing epileptogenesis is unproven. There is an urgent need for prospective, high-quality studies to guide the duration and selection of ASM therapy in this highrisk population.

Such a distinction between structural versus systemic etiologies may also facilitate more focused clinical and translational studies, allowing a better understanding of chronic seizure risk and the mechanisms that drive epileptogenesis. Ultimately, this approach may contribute to identifying interventions capable of modifying epileptogenesis in patients at higher risk.
